# Antimicrobial Use in Dromedary Camels: Off-Label Practices, Welfare Implications, and Antimicrobial Resistance Within a One Health Framework—A Narrative Review

**DOI:** 10.3390/vetsci13090959

**Published:** 2026-09-14

**Authors:** Aftab Shaukat, Illahi Bakhsh Marghazani, Abdulrahman A. Alkheraif, Mohamed Tharwat

**Affiliations:** 1 Animal Science Division, Department of Modern Agriculture, College of Life and Environmental Sciences, Hunan University of Arts and Science, Changde 415000, China; dr.aftabshaukat@huas.edu.cn; 2 Faculty of Veterinary and Animal Sciences, Lasbela University of Agriculture, Water and Marine Sciences (LUAWMS), Uthal 90150, Pakistan; ibmarghazani@luawms.edu.pk; 3 Department of Pathology and Laboratory Diagnosis, College of Veterinary Medicine, Qassim University, P.O. Box 6622, Buraidah 51452, Saudi Arabia; 4 Department of Clinical Sciences, College of Veterinary Medicine, Qassim University, P.O. Box 6622, Buraidah 51452, Saudi Arabia

**Keywords:** antimicrobial resistance, dromedary camels, off-label practices, One Health, zoonosis

## Abstract

*Camelus dromedarius* (the dromedary camel) plays a vital role in the lives, food security, and culture of pastoral people in many arid and semi-arid areas of Africa, the Middle East, and South Asia. Despite their economic and nutritional value, camels are poorly represented in veterinary pharmacology, with limited numbers of antimicrobial agents formally licensed for use in this species. These practitioners and owners therefore depend heavily on off-label and extra-label administration of drugs developed and dosed for cattle, small ruminants or humans. This narrative review synthesizes published work related to antimicrobial use in dromedary camels, highlighting the pharmacokinetic uncertainties of extrapolating doses from other species. This review distinguishes between direct dromedary data and indirect evidence from other camelids, as well contextual practices within mixed livestock systems to fill evidence gaps. It addresses the non-clinical aspect of suboptimal antimicrobial use, such as under-analgesia, irrational dosing and untimely or off-formal treatment. It also discusses the ever-expanding literature on antimicrobial-resistant bacteria found in camel-derived products, camel ticks and camel feces, such as the presence of ESBL and MDR *E. coli*, *Klebsiella pneumoniae*, and *Staphylococcus* spp. The findings are discussed in the context of the One Health paradigm, and possible routes of camel-to-human exposure through raw milk and meat, as well as environmental dissemination of resistance genes, are recommended for surveillance and stewardship.

## Simple Summary

Dromedary camels are an important source of food, income, and cultural heritage for millions of people living in arid and semi-arid regions of Africa, the Middle East, and South Asia. However, compared with other food-producing animals, camels have received relatively little attention in veterinary medicine. Very few antimicrobial drugs are specifically licensed for use in camels, meaning that veterinarians and livestock owners often rely on medicines and dosage regimens developed for other animal species. These off-label practices may result in ineffective treatment, poor animal welfare, drug residues, and the emergence of antimicrobial-resistant bacteria. This review summarizes the current knowledge on antimicrobial use in dromedary camels; the challenges associated with extrapolating treatments from other livestock species; and the evidence for antimicrobial-resistant bacteria reported in camel milk, meat, feces, and ticks. It also discusses how resistant bacteria and resistance genes may spread between camels, humans, and the environment through food production systems and close human–animal interactions. By highlighting existing knowledge gaps and the need for improved antimicrobial stewardship, camel-specific pharmacological research, and integrated surveillance within a One Health framework, this review emphasizes the importance of including dromedary camels in global efforts to combat antimicrobial resistance and protect both animal and public health.

## 1. Introduction

*Camelus dromedarius*, the dromedary camel, is a single-humped ungulate uniquely adapted to hot arid and semi-arid environments, with an estimated population of more than thirty-five million in the world, with the majority in East Africa, the Horn of Africa, the Arabian Peninsula, and South Asia. The camel is a useful animal for transporting goods; it is a source of milk, meat, hide and fiber for millions of pastoralists and agro-pastoralist households, and camel milk is now a growing commodity across the Middle East, North Africa and parts of Asia [1]. The dromedary camel is a valuable alternative protein and income source that can survive and thrive in many arid rangelands where cattle and small ruminants are at risk of death during prolonged drought [2]. The camel’s importance is likely to increase further with increased climate variability in these areas [3].

However, veterinary pharmacology in relation to the dromedary camel is rather underdeveloped, considering its increasing economic and nutritional value [4]. Very few antimicrobial, analgesic, anti-inflammatory medications have been specifically evaluated and licensed in camels, and dosing is often extrapolated from cattle, small ruminants, horses, or even human medicine [5]. This practice, which is often referred to as “off-label” or “extra-label” use is common among minor and food-producing species. In such cases, the anatomical, physiological and metabolic characteristics of the animal might differ significantly from those of the species for the dosage was designed [6]. Due to the unique physiology of the camel, its digestion is unusual, with a three-chambered forestomach and different absorption and elimination rates of drugs, which may lead to differences in drug action compared to cattle, as well as concerns about subtherapeutic dosing [7].

The exposure of subtherapeutic or inconsistent antimicrobials is widely recognized as an important factor in the selection of antimicrobial resistance [8]. In surveys carried out in the Arabian Gulf, East Africa and North Africa, camels and camel products have been increasingly found to carry antimicrobial-resistant and multidrug-resistant bacteria, such as extended-spectrum beta-lactamase producers [9]. Meanwhile, the welfare aspect of extrapolated and sometimes informal treatment of antimicrobial and analgesics in camels has been comparatively poorly researched, even though inappropriate pain relief, incorrect dosages and delayed access to qualified veterinary care are all recognized welfare issues in camelid medicine in general.

This review covers published peer-reviewed research that includes camel therapeutics, camel production system surveys and microbiological investigations of camel derived samples, derived from evidence in East Africa, the Horn of Africa, the Arabian Gulf, North Africa and South Asia. Similar evidence from other minor and food-producing species, such as cattle, small ruminants and llamas and alpacas (South American camelids), is also included where it elucidates the principles of pharmacokinetics and/or regulatory patterns that are likely to apply to the dromedary camel, in view of the general paucity of camel primary data in several therapeutic areas. Although the literature is rich with works focusing on the prevalence of AMR in camels, this narrative review provides a novel synthesis by explicitly connecting these microbiological findings with the underlying drivers: gaps in camel pharmacokinetics, welfare issues of off-label prescribing, and real challenges of unsupervised pastoral antimicrobial use. The goal is to combine this scattered evidence to inform evidence-based antimicrobial stewardship and animal welfare policies and contribute to regulatory decision making in dromedary camels, a species of fundamental importance to global food security that has received comparatively limited attention in veterinary pharmacological research.

## 2. Methodology of the Narrative Review

A literature search was done on PubMed, Scopus, Web of Science and Google Scholar to synthesize the present knowledge of antimicrobial use and antimicrobial resistance in dromedary camels. Search terms were used in various combinations, for example, “dromedary camel”, “*Camelus dromedarius*”, “antimicrobial resistance”, “pharmacokinetics”, “off label”, and “One Health”. Only English-language, peer-reviewed articles were included with no restriction on the date of publication but with a focus on the last 10 years. The primary pharmacological data is limited in dromedary camels; hence, a structured evidence-grading scheme was used to classify the findings mentioned in this review.

## 3. Antimicrobial Use Practices in Dromedary Camel Production Systems

**Figure 1:**
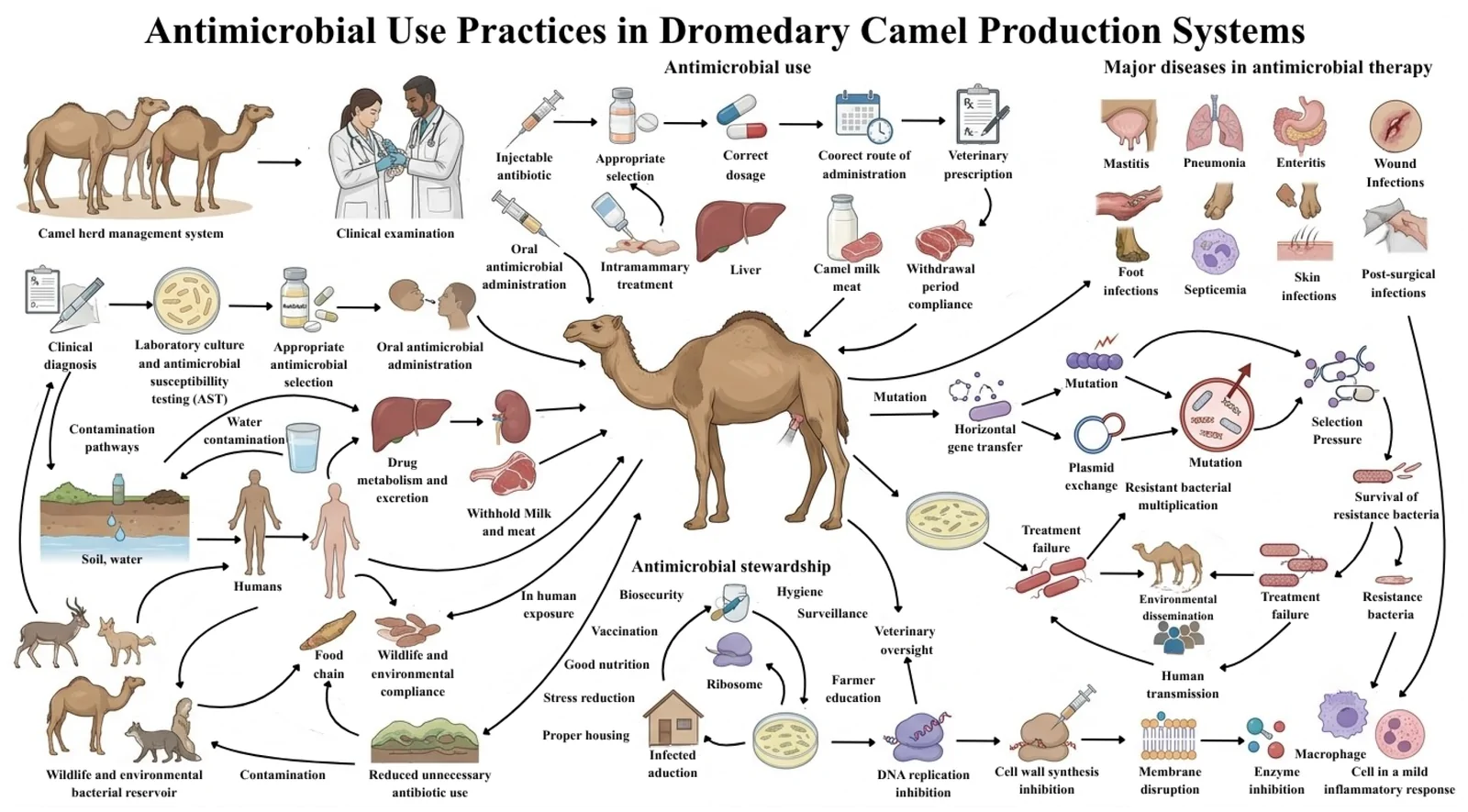
Conceptual framework: Use of antimicrobial drugs in dromedary camel production systems. This schematic shows the complicated system of veterinary interventions initiated from clinical diagnosis to the application of different antimicrobial drugs (injectable, oral, and intramammary) for the treatment of major camel diseases, including mastitis, pneumonia and enteritis. It emphasizes the need to strictly follow the withdrawal periods for drug residues in milk and meat, risks of treatment failures, antimicrobial resistance selection, and the downstream risks of the dissemination of antimicrobial residues in the environment when antimicrobial stewardship principles are not followed.

The use of antimicrobial medicines in dromedary camels is largely in pastoral and semi pastoral farming systems, where there is low a level of formal veterinary infrastructure in the extensive systems (Figure 1) [10]. Many of the diseases that are treated in camels are like those that are treated in cattle and small ruminants in the same herd, such as bacterial pneumonia, mastitis, wound infections, diarrheal disease, tick-borne conditions and infections of the reproductive tract [11]. The classes of medication reported in camel herds in East Africa, the Horn of Africa and the Arabian Peninsula are like those reported in other food-producing species in low- and middle-income countries, with broad-spectrum agents being the most common classes [12].

Milk production from camels has been rapidly growing as a commercial business in the GCC countries and other countries and has been paralleled by an increasing use of antimicrobials to control mastitis and other infections in lactating animals [13]. Bacterial isolates with multiple resistance to antimicrobial agents have been reported from raw camel milk sold in retail outlets in several countries in the Gulf region, suggesting a possibility of antimicrobial exposure on the farm. However, because source-tracking data are limited, these findings could also result from multisource contamination occurring during on-farm milking, processing, transportation, or retail handling. In several regional surveys, the presence of antimicrobial residues and resistant bacteria have also been linked to the production of camel meat and camel tick management [14].

In mixed pastoral systems, such as a comparative survey of mastitis pathogens and associated antimicrobial resistance in mastitis samples from dairy cows, camels and goats in two pastoral districts in Southern Ethiopia [15], the presence of bacterial mastitis pathogens has been documented in camel alongside the other dairy species. This suggests that camel studies may be applying empirical therapeutic regimes similar to those applied to other dairy animals, but further data on formal prescriptions or records of medications used on the farm would be needed to prove this [16]. These results indicate that camel-specific mastitis management is often not significantly different to that of co-habiting animals, despite the physiological differences that could have an impact on drug activity in camels [17].

The distribution of antimicrobials in camel-keeping communities is often outside the veterinary system [18]. Many pastoralists in East Africa and the Horn of Africa go to agrovet stores or informal drug vendors to buy antimicrobials without a prescription or veterinary advice, as has been well reported with respect to cattle, sheep and goats across the region [19]. It is likely to be the same with camels, as many of these species are managed together in mixed herds [20]. In several studies, pastoralists reported being expert guardians of the knowledge of veterinary drugs and treating their livestock without a laboratory diagnosis or veterinary advice, instead relying on experience [21]. This means that there is no specific dosing information available for camels, and with this, the door is open to the off-label and extra-label practices that are detailed in the next section [22].

## 4. Off-Label and Extra-Label Drug Use: Pharmacokinetic and Regulatory Gaps

The use of veterinary medicine outside of the approved label use is known as off-label or extra-label use, terms whose legal definitions and permissible conditions vary significantly across global jurisdictions and may involve deviations in target species, dose, route, frequency, or indication [23]. Antimicrobials are commonly used off label, as there is a lack of camel-specific licensing and literature on withdrawal [24]. The camel literature has repeatedly reported that there is a lack of specific dosing recommendations for camels and that dosing is generally extrapolated from cattle, small ruminants, horses or even human medicine [14].

There are significant pharmacokinetic concerns with this extrapolation [25]. Camels have a physiologically different three-chambered forestomach, longer gastrointestinal transit times and body water distribution than true ruminants [26]. Primary pharmacokinetic studies in camels tend to show different parameters, such as differences in the elimination of half-life, volume of distribution and clearance of some antimicrobials between cattle and camel, which make it unsafe to directly use dosage regimens derived from cattle. For instance, pharmacokinetic studies have shown that dromedary camels have a significantly longer terminal elimination half-life of oxytetracycline (about 46.1 h) compared to cattle, which have a half-life of 6–9 h [27]. Oral bioavailability and elimination kinetics of drugs like meloxicam have been investigated in camelids (llamas and alpacas), and it has been shown that there can be significant differences between camelids and ruminants. Although pharmacokinetically analgesic drugs cannot be swapped for antimicrobials without further caution, the present results point to a general physiological fact: absorption and elimination mechanisms in camelids significantly differ from those in true ruminants, a difference that is of particular concern when considering the extrapolation of drugs within the same pharmacological class. Veterinary guidance states that, if possible, these should be extrapolated from other camelid species, not ruminants [28]. A similar species-specific absorption, distribution, and elimination profiles of antibiotics compared to those in cattle were also found in early pharmacokinetic studies performed in camels, suggesting that dosing regimens cannot be taken verbatim from bovine medicine and that drug levels in camels must be obtained [29].

There are two main repercussions with respect to this pharmacokinetic unknown, both of which currently represent plausible theoretical risks rather than confirmed field outcomes due to the lack of dromedary-specific residue surveillance [30]. First, there is the possibility of extrapolating doses from other species and not achieving subtherapeutic levels in camels, which, in turn, may lead to incomplete eradication of bacteria, longer duration of illness and development of resistance [31,32]. Secondly, doses can be theoretically excessive, leading to toxicity, undesirable side effects and drug residues being introduced into the human food chain (milk and meat) [33]. The withdrawal periods for the most used antimicrobial drugs in camels are not clearly determined, and farmers and vets often rely on withdrawal periods that were determined in cattle [34]. This may not accurately reflect those in camels and could lead to drug residues in the milk or meat of camels for a longer period than is justifiable [35].

The route of administration is an additional factor that is not taken into consideration in camel practice [36]. Camelid oral drug administration (continuous) should be avoided with many oral preparations; for example, some antibiotics, gastric protectants, and other preparations intended for the ruminant forestomach should not be used orally in camelids, and other drugs that may be effective via oral administration in other species may be ineffective [22]. Especially in countries where injectable medication products are not as readily available or more expensive than oral products, clinicians who are unaware of this distinction may end up choosing a route of administration that will not support therapeutic success, thereby leaving the gut microbiota exposed to subtherapeutic levels of drugs that can exert selective pressure for resistance.

These are exacerbated by regulatory frameworks. In many camel-rearing countries, the national veterinary drug registration schemes do not specifically focus on the case of camels [37,38]. However, antimicrobial products are registered, marketed and labelled according to the specifications for cattle, sheep, goats or poultry, thereby limiting the camel owner and veterinarian in their available authoritative guidance [39]. It is the same if the camelids are in a high-income setting like the United States, where no antimicrobial or antianalgesic drugs have been approved by national regulators for camelids and vets must use drugs off label to treat camelid patients, even in a well-resourced clinical system. In early pharmacological surveys, the peculiarities of the camel—anatomical, physiological and biochemical—were concluded to require focused investigations with respect to therapeutic use of antibiotics in camel production systems [40]. Although this call was only partially answered, the lack of evidence available to contemporary camel users has resulted in a poor evidence base on the basis of which to make dosing decisions [41].

## 5. Drivers of Off-Label and Irrational Antimicrobial Use

There are several related factors contributing to the irrational and off-label use of antimicrobial agents in the camel-keeping community [42]. In remote and arid rangelands, veterinary services may be scarce [43]. Herders are often the first to react to sick animals, relying on what they have learned informally through experience and over time and not on formal education [44]. However, in pastoralist households in East Africa and beyond, high rates of self-administered antimicrobial treatment, short treatment durations and insufficient knowledge of the appropriate duration for the withdrawal period after treatment have been reported [45]. This is likely to be the case for camel-keeping households, where camel and livestock (cattle and small ruminants) are kept together under one management system [46].

These practices are largely driven by severe economic constraints [47]. The use of antimicrobials has been recognized as one of the available and relatively low-cost ways to minimize losses [48]. A large proportion of livestock surveys indicated that a large proportion of respondents used antimicrobials when a diagnosis of disease had not been made—more to promote growth or milk production potential [49]. The informal agrovet shops (IVS), which are also responsible for the distribution of antimicrobials without prescription, are not well regulated [50]. This makes antimicrobials available, even when they are not officially recommended or used in treatment [51]. There is limited data on the direct use of antimicrobial agents in camel-based systems; however, data from mixed-species pastoral systems are relevant. Survey results on the unsupervised use of antimicrobials in these areas of cattle, sheep and poultry indicate widespread unsupervised use [52,53]. The co-management of these species strongly implies human-like drivers for these species as well, but these results cannot be directly attributed to camel use. Similar problems of uncontrolled dosages, human formulation of animal products, and insufficient veterinary supervision have been reported in other studies (Table 1) [21].

Comparative data for the region illustrate the degree of embedding of these behaviors in food animal industries [54]. These sectors have herders and drug outlets in common with those of camel-keeping communities and share regulatory frameworks [55]. Results from a survey indicated that most dairy-cattle keepers self-prescribed antimicrobials for their animals in north–central Nigeria [56]. Many of them used antimicrobials without a diagnosis, and some used antimicrobials to increase milk production instead of for any diagnosed disease [57]. A proportion of 68% of the respondents did not know what was meant by the use of antimicrobials in a way that is not appropriate [58].

Similarly, a cross-sectional survey of poultry farmers in rural areas of Punjab, Pakistan, indicated that colistin sulphate, an antimicrobial drug of last resort for human use, and amoxicillin trihydrate were the most used antimicrobials [59], often used without veterinary advice, with the frequency of use found to vary seasonally based on the perceived presence or lack of a specific poultry disease [60]. These studies provide documentation of access to and use of antimicrobials in unsupervised settings in the context of livestock (cattle and poultry) [61]. They were conducted in the same mixed farming/pastoral areas where antimicrobials are used for camels [62].

Irrational use is also significantly due to diagnostic limitations [63]. Treatment is usually presumptive based on clinical signs only, and broad-spectrum drugs are often used [64]. There is limited laboratory culture and susceptibility testing capacity in most camel-producing areas [65]. The use of a drug might be based on the potential of an unidentified cause [66]. Such drugs are used at doses that may not be calibrated to the infecting organism and without evidence that the agent is even suitable for the presumed infecting organism [67,68].

**Table 1:** Reported antimicrobial use practices, off-label extrapolation, and regulatory status in camel-keeping and comparable regional livestock systems.

Evidence Category	Region/Country	System/Species Context	Antimicrobial Agent or Practice	Reported Prevalence or Finding	Key Observation	References
Indirect Camelid Evidence (South American)	General camelid clinical guidance	Camelid medicine broadly, including dromedary and South American camelids	Route of administration for oral drugs	Many oral drugs fail to survive passage through the three-chambered forestomach	Parenteral administration is frequently required where oral dosing would be used in monogastric species	[22]
Contextual Livestock Evidence	Pakistan, rural Punjab	Poultry farming households	Colistin sulphate and amoxicillin trihydrate	Used by 60% of surveyed participants	Highlights unsupervised access to last-resort human antimicrobials in regional veterinary markets	[59]
Contextual Livestock Evidence	North–central Nigeria	Pastoral dairy cattle households (mixed herd context)	Self-prescription of antimicrobials (Tetracycline, Penicillin, etc.)	Reported by 61.7% of respondents; 67.4% applied without diagnosis	Demonstrates severe antimicrobial misuse drivers in analogous pastoral systems	[69,70,71,72,73]
Direct Dromedary & Mixed Herd Evidence	Ethiopia, three agroecological zones	Smallholder and pastoralist livestock keepers; mixed livestock including cattle, sheep, goats, and camels	Tetracyclines, aminoglycosides, and trimethoprim-sulfonamide	Highly prevalent; frequent under-dosing and over-dosing	Antimicrobial usage behaviors observed in herds where camels are co-managed with ruminants	[74,75,76,77,78,79,80]
Contextual Livestock Evidence	Kenya	Maasai pastoralist households; cattle, sheep, and goats	Tetracycline; penicillin–streptomycin	Used by 65% and 28% of surveyed pastoralists, respectively	Highlights preferred empirical treatments in East African pastoral settings	[81,82]
Contextual Livestock Evidence	Tanzania	Poultry and pig smallholder farmers	Antimicrobial use for growth promotion	Reported by 26% of surveyed farmers	Illustrates non-therapeutic use motives in regional food animals	[83]
Contextual Livestock Evidence	Rwanda	Livestock farming households	Penicillin–streptomycin combination	Reported by 100% of respondents	Universal reported use of this combination for general animal treatment	[84]
Indirect Camelid Evidence (South American)	United States	Camelid veterinary practice; llamas and alpacas	Formal drug licensing status	No antimicrobial or analgesic drugs are approved by national regulators specifically for camelid species	Extra-label prescribing under general veterinary authority is routine practice	[85]
Indirect Camelid Evidence (South American)	Camelid pharmacology literature	Experimental pharmacokinetic study; six adult llamas	Meloxicam; oral and intravenous administration	Oral dose of 1 mg/kg and IV dose of 0.5 mg/kg tested	Demonstrated bioavailability and elimination kinetics distinct from cattle data used for extrapolation	[86]
Direct Dromedary & Mixed Herd Evidence	Historical camel pharmacology survey	Camel therapeutics literature review	Species-specific dosage recommendations	Described as rare across the surveyed literature	Dosing in clinical practice is typically extrapolated from cattle, small ruminants, or other species	[87]

## 6. Welfare Implications of Off-Label Antimicrobial and Analgesic Use

Extrapolated and informally given antimicrobial treatment in dromedary camel has a multifaceted welfare impact [41]. If dose-rate regimes for cattle or other species are not proven to have subtherapeutic value in camels, they may be suffering from prolonged untreated or inadequate treatment, which could involve pain, sick status and delayed recovery [88]. Pneumonia, mastitis and wound infection, if not resolved in an adequate fashion due to underdosing, can develop into more painful disease states and add to animal suffering that could have otherwise been prevented by adequate treatment [89].

The same limitations apply to camelids in terms of using analgesic and anti-inflammatory drugs. The use of antimicrobial and/or analgesic drugs is specifically addressed in reviews of pain management in camelids [90]. There is a warning that the use of these drugs in this species is extrapolated from cattle, sheep, and/or horse use [91]. There are documented differences in bioavailability and elimination of these drugs between camelids and these other species [92]. Camels may suffer from inadequate analgesia for painful procedures and for painful disease processes, particularly with the increasing use of camelids as companion animals in addition to their use as production animals [90].

The magnitude of this disparity is demonstrated by the medicines that can be used for pain relief [93]. In camelid medicine in general, transdermal fentanyl patches and injectable NSAIDs are occasionally used for pain control [94]. However, the dosing intervals, absorption times, and durations of action for these products have typically been determined in other species and extrapolated to camelids [95]. There is limited data to validate their analgesic efficacy and/or safety in camelids [96]. Minimal or no analgesic support may be given during painful procedures such as wound debridement, obstetric intervention and management of traumatic injury [97]. This situation is not reported by production-focused veterinary surveys and directly impacts the wellbeing of animals [98].

A further welfare risk is that of overdose because of inappropriate extrapolation [99]. High levels of a drug may cause direct toxicity, gastrointestinal upset or organ-specific toxicity [100]. On the other hand, undertreatment because of cost-cutting may result either from reduced dosage (dilution) or the use of an inferior course of treatment (fewer doses of less effective drugs) [101]. This extends the length of time a patient is ill and reduces the benefit of antimicrobial treatment to the patient’s welfare [102]. Pastoralist antimicrobial practice studies have revealed high percentages of livestock owners using more or less than recommended doses [82]. They were found to be stopping antibiotics too soon or too late and not knowing the clinical effects of these practices [103]. These practices are often used in parallel informal veterinary services, but other veterinary measurements need to be developed specifically for camels, including pain scoring and monitoring of disease duration, to definitively quantify the resulting animal suffering [104].

In addition to animal welfare, the consequences at the herd level should also be considered [105]. A lack of consistent or formal antimicrobial use may result in disease going undetected [106]. This ensures that clinical signs are not reduced but, rather, the disease is covered up, delaying disease outbreak recognition within a herd [107]. This perpetuates spread amongst in-contact animals [108]. The lack of reliable camel-specific clinical and pharmacological guidance therefore not only poses a problem for camel production systems but also for animal welfare [109].

## 7. Antimicrobial Resistance in Dromedary Camels

There is increasing evidence published on the presence of antimicrobial-resistant bacteria in dromedary camels and their derived products from the Arabian Gulf, North Africa and East Africa [110]. The presence of multidrug resistance (MDR) among recovered bacterial isolates (not the milk samples) from retail outlets in Kuwait was found in approximately 80% of the samples in a study examining raw camel milk, including pathogens of *Klebsiella pneumoniae*, *Escherichia coli* and *Enterobacter hormaechei* with transferable resistance genes, both by culture-based and by shotgun sequencing methods [111]. This study implies a possible pathway of public health exposure as camel milk is consumed in the region raw, although epidemiological studies in this regard are required to establish direct association with human infections caused by these isolates [4].

**Figure 2:**
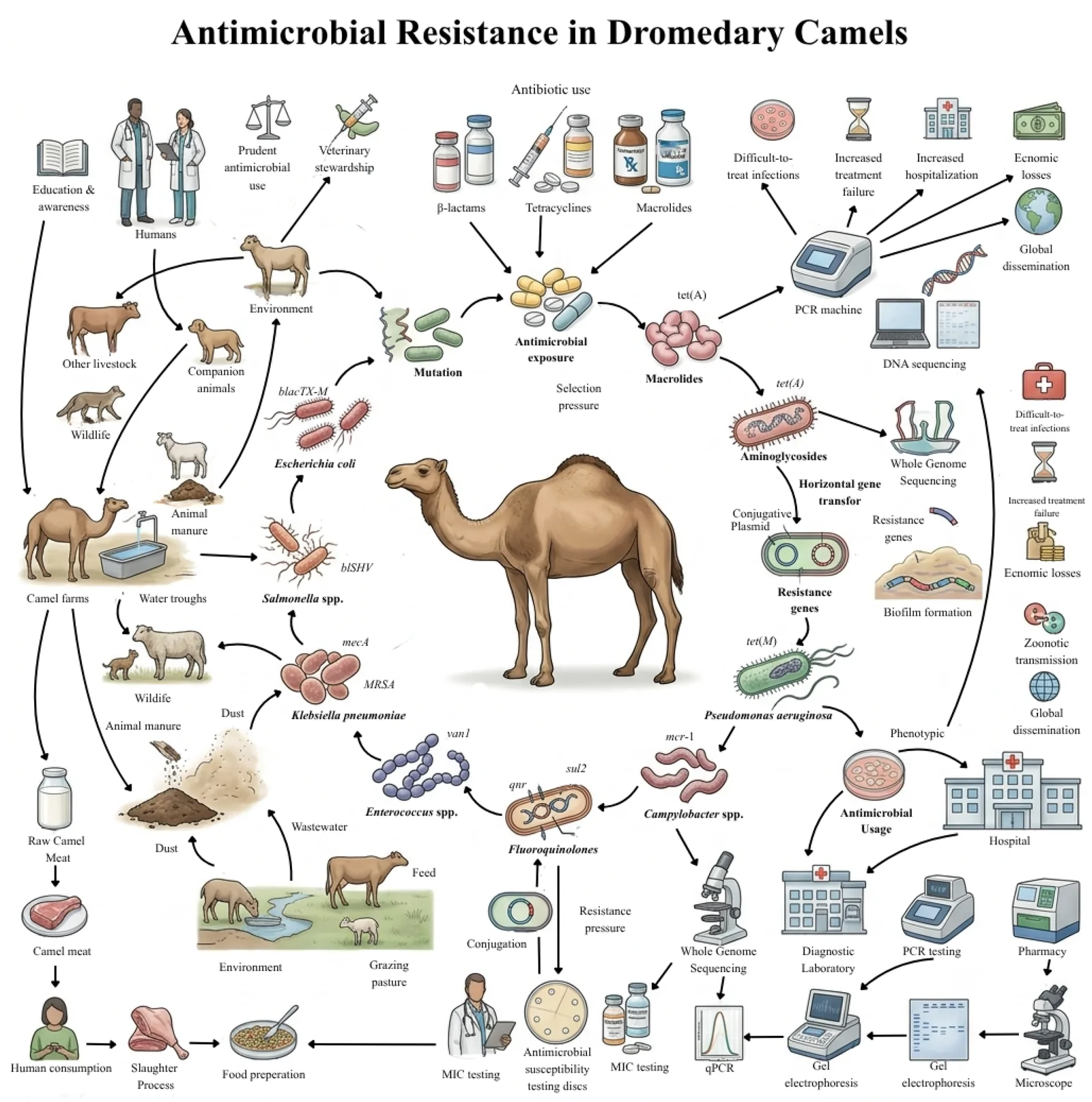
Conceptual framework: Development of antimicrobial resistance in dromedary camels. The diagram illustrates biological and ecological mechanisms that contribute to antimicrobial resistance in camel populations. It describes the selection pressure that is imposed by exposure to key classes of antibiotics (such as β-lactams, tetracyclines and macrolides), resulting in resistance arising through genetic mutation and horizontal gene transfer. The figure also shows the clinical and economic impacts of resistant infections and how genomic sequencing and diagnostic laboratory surveillance can help to identify resistant determinants in the veterinary and healthcare environment.

Similarly, antimicrobial resistance of *Escherichia coli* has been found in dromedary camels in Africa, with tetracycline, ampicillin, streptomycin and sulfamethoxazole trimethoprim combinations being most detected and a high proportion of resistant isolates identified as multidrug-resistant [112]. ESBL-producing *E. coli*, specifically those harboring the CTX-M-15 enzyme encoded by the blaCTX-M gene, have also been isolated from apparently healthy camels, suggesting that ESBL-producing bacteria can occur sub-clinically in camels without clinical symptoms (Figure 2).

A new and scarcely recognized reservoir is Camel ectoparasites [113]. The characterization of bacteria found in ticks (*Hyalomma dromedarii* and other tick species) on camels in Saudi Arabia yielded a range of multidrug-resistant Gram-positive and Gram-negative isolates, including some that resist cephalosporins and other clinically important agents [114]. However, not all the isolates derived from the ticks were multidrug-resistant against three or more classes of antimicrobial agents [115]. The results indicate that camel ticks exhibit carriage of resistant bacteria; however, direct vector competence and transmission pathways remain to be conclusively demonstrated [116].

More than twenty species of bacteria were isolated from ticks collected from camels from the Al-Jouf region of Saudi Arabia, including dozens of isolates from *Hyalomma excavatum* [117]. *Hyalomma dromedarii* were identified, including over two dozen that were Gram positive; resistance was found for benzylpenicillin, oxacillin, clindamycin, vancomycin, and rifampicin among Gram-positive isolates, as well as for cefoxitin, ampicillin, and ceftazidime [114]. A few cephalosporins were identified among Gram-negative isolates, but susceptibility to other agents such as linezolid, gentamicin, and several fluoroquinolones remained largely intact [118]. The evidence base suggests that there is no country or sample type where camel-associated antimicrobial resistance is limited to just one sample type or just one country [119]. However, that it is shared by milk, feces, and ectoparasites across multiple continents, in addition to samples from mixed pastoral herds in Southern Ethiopia [120]. It is important to note that the prevalence rates are not comparable among these studies (summarized in Table 2). There are also considerable methodological variations reported in the literature, such as different bacterial isolation methods, variable definitions of multidrug resistance (MDR), and different standards for antimicrobial susceptibility testing (AST) (e.g., CLSI vs. EUCAST). These percentages illustrate a general trend of resistance emerging but do not serve as epidemiological guidelines.

A recent synthesis of the reported prevalence of ESBL production in AMR isolates from camels reported a wide variation in ESBL prevalence from study to study while highlighting the presence of carbapenemase genes and plasmid-mediated colistin resistance determinants in ESBL-producing camel-associated isolates in the Gulf Cooperation Council and North Africa region [121]. The same synthesis identified similarities in molecular features of resistance genes recovered from camel isolates with those found in human clinical isolates. This molecular similarity raises the possibility of epidemiological links between dromedary camels and humans, but more genomic epidemiology is needed before definitive epidemiological links can be established [122]. Furthermore, camel production systems are not well represented in national antimicrobial resistance surveillance and stewardship programs relative to cattle, poultry and swine [123].

However, dromedary camels have been of interest to scientists, especially with respect to resistance as a source of new antimicrobial peptides [124]. Even though camel antimicrobial stewardship is not as well developed as in other livestock, researchers have identified camel-derived cathelicidin peptides [125] such as those with activity against methicillin-resistant *Staphylococcus aureus* and multidrug-resistant *Escherichia coli* in laboratory studies, suggesting alternative therapeutic paths for the camel innate immune system [126].

**Table 2:** Reported antimicrobial resistance findings in dromedary camels and camel-associated samples.

Region/Country	Sample Source	Key Bacterial Isolates	Summarized Antimicrobial Resistance Findings	Additional Notes	References
Kuwait	Raw camel milk (retail outlets)	Mixed Gram-negative (*K. pneumoniae*, *E. coli*, and *E. hormaechei*)	~80% of recovered isolates were multidrug-resistant (MDR). Highest resistance observed against penicillins, tetracyclines, and carbapenems.	Shotgun sequencing confirmed transferable AMR genes.	[111,121,127]
Africa (Multi-country)	Camel feces	*Escherichia coli*	Isolates demonstrated frequent resistance to tetracycline (11.7%), ampicillin (10.5%), and streptomycin (10.5%).	85% of ampicillin-resistant isolates were MDR. Low prevalence (0.6%) of ESBL-producing *E. coli* (blaCTX-M-15) also detected.	[112,128,129]
Saudi Arabia (Hail & Al Jouf Regions)	Camel ticks (*Hyalomma dromedarii* and *H. excavatum*)	Gram-negative and Gram-positive isolates	Gram-negatives showed high resistance to cefazolin (100%), cefoxitin (75%), and ceftazidime (62.5%). Gram-positives showed high resistance to benzylpenicillin (100%) and oxacillin (93.3%).	Isolates remained broadly susceptible to higher-generation cephalosporins, linezolid, and fluoroquinolones.	[114,117,118,130]
Southern Ethiopia	CMT-positive milk (mastitis screening)	*Escherichia coli* and *Staphylococcus aureus*	High resistance reported across multiple classes, including spectinomycin, vancomycin, and doxycycline.	Most recovered *S. aureus* isolates were classified as MDR. Sampled alongside dairy cows and goats.	[15,131]
Egypt	Camel meat	*Escherichia coli* (Shiga toxigenic)	High rates of extended-spectrum and multi-drug resistance patterns.	Demonstrates contamination risk in camel meat supply chains.	[119]
GCC & North Africa	Pooled camel-associated isolates	Mixed Gram-negative organisms	Documented presence of ESBL production, carbapenemase genes, and plasmid-mediated colistin resistance (mcr-1).	Molecular similarity to human clinical isolates indicates a shared resistance reservoir.	[132,133]

## 8. One Health Dimensions: Zoonotic Transmission and Environmental Spread

**Figure 3:**
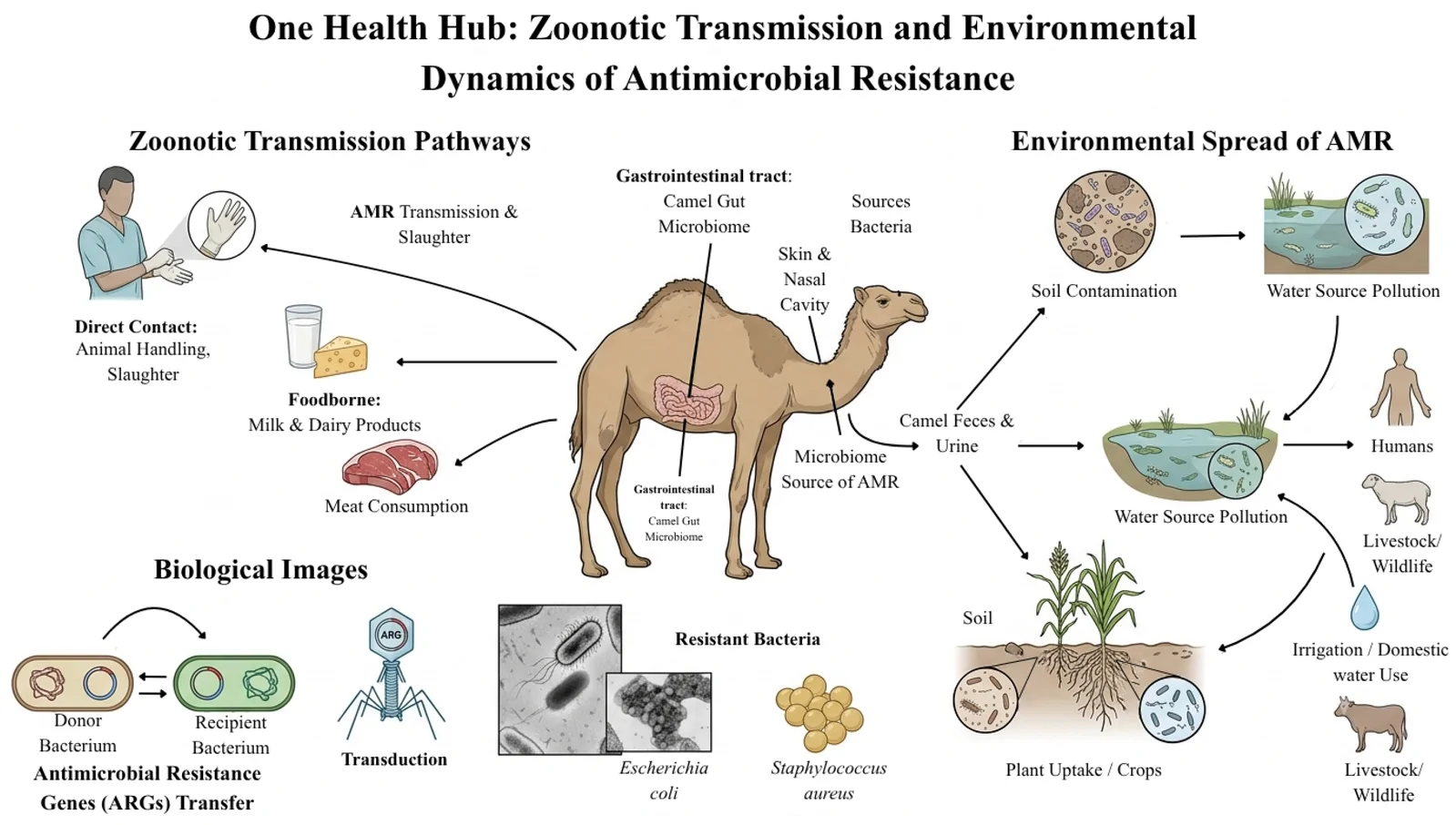
Conceptual framework: Hypothesized zoonotic transmission and environmental dynamics of antimicrobial resistance in dromedary camels. This One Health model shows the possible pathways by which AMR bacteria and genes could be transmitted from the dromedary camel microbiome, emphasizing the possibility of zoonotic transmission from direct handling of animals and from raw milk and meat. In addition, it outlines the conceptual spread of AMR by camel feces and soil contamination and the subsequent pollution of water sources that can ultimately affect co-grazing livestock, wildlife and human communities.

The One Health concept, encouraged by international human health, animal health, food and environmental agencies, has resulted in the understanding that human, animal and environmental health are interlinked and defined by an inter-sectoral approach, especially for an inter-sectoral threat like AMR (Figure 3) [134]. The emerging evidence of antimicrobial resistance associated with camels provides a highly relevant yet under-investigated model for this framework [135]. Camels encounter humans through direct contact, as well as ingestion of raw meat and milk products, living in the same environments in pastoral areas, with several potential pathways for the transfer of resistant bacteria and resistance genes between camels and their human owners [136]. Consumption of raw camel milk has been documented and is culturally significant throughout the Arabian Peninsula, the Horn of Africa and parts of South Asia and is therefore considered a plausible route of exposure to resistant organisms for humans [4].

In addition, there is a broader zoonotic disease picture associated with the use of antimicrobials and resistance in camels [137]. Food-borne bacterial risks have been reported with camel milk, meat and through direct contact with camels, including with respect to Salmonella, *Escherichia coli*, Staphylococcus and Campylobacter; other camel-associated zoonoses such as bruellosis have also been noted [138]. The selective pressure for resistance is increased for both camels and affected humans when these infections are treated with broad-spectrum antimicrobials on an empirical basis within both populations [129].

This transmission web can be extended by the environmental compartments. Resistant bacteria and antimicrobial residues excreted by treated camels can be spread to other livestock, wildlife, and human communities in arid rangelands via contaminated water sources and soil [139]. However, paired environmental-sampling and source-attribution studies are currently lacking and are necessary to confirm these specific transmission routes. Transmission routes via camel ticks and other ectoparasites that can carry resistant organisms from one host to another should also be investigated [114]. Although there is a biological possibility that resistant bacteria in camel populations could be disseminated to co-occupying livestock (cattle, sheep, and goats) and humans via the shared environmental reservoirs, structured source-attribution studies with large numbers of samples are required to determine the extent of this environmental dissemination [140].

Camel production systems are surprisingly not well represented in any national or international antimicrobial resistance surveillance program and have been prioritized less than cattle, poultry, and swine production systems [141]. The lack of surveillance hinders our understanding of the extent to which the resistance arises from camels and prevents public health and veterinary officials from developing targeted interventions, despite the mounting evidence indicating that camels are an important but largely uncharacterized reservoir of resistance in the One Health antimicrobial resistance web [142].

## 9. Surveillance Gaps and Stewardship Recommendations

There is a need for coordinated action on several fronts to tackle the issue of off-label antimicrobial use, as well as the welfare implications and the risks for antimicrobial resistance in dromedary camels [24]. Specific pharmacokinetic and pharmacodynamic studies should be performed in dromedary camels to determine species-specific dosing regimens, as well as withdrawal and analgesic protocols, and not simply extrapolate from cattle or other camelid species [143]. This evidence gap has been identified as a priority, and closing it has been consistently identified as a target to reduce therapeutic uncertainties and welfare impacts in current extrapolated practice [144].

Antimicrobial resistance surveillance of camels should be formally established as part of national and regional One Health monitoring programs, in addition to the other One Health sectors, which are currently the focus of most surveillance efforts—namely, cattle, poultry and swine [145]; standardized susceptibility testing; and, if possible, genomic characterization of resistance determinants in all sampling points of camel milk, meat and feces [111]. In practice, surveillance programs should prioritize antimicrobials that are of critical importance in human medicine (such as higher generation cephalosporins and carbapenems), major camel pathogens and key sampling points, ranging from farms to the retail level. Associated ectoparasites would provide useful information to determine the actual prevalence and public health importance of camel-associated resistance across various production systems and regions [120].

Better access to trained veterinary health workers and the capacity for diagnostics in community of camel keepers, especially in pastoral rangelands in remote areas, would help to minimize the use of informal drug vendors and self-diagnostic and self-treatment services [146]. In other livestock sectors, training courses that are based on existing practical knowledge of pastoralists and focus on accurate knowledge of correct dosage, treatments and length of withdrawal have proven to be effective; these courses could be tailored specifically for camel-keeping households [18].

Regulatory bodies in camel-producing countries should explicitly take camel into account when developing national veterinary drug registration and antimicrobial stewardship policies [147]. This would lay the groundwork for camel-specific product labeling, evidence-based guidance for prescribing, and mandatory withdrawal periods. Measurable indicators need to be set in order to track stewardship progress. These should include the percentage of herders who complete a treatment course, antimicrobial susceptibility testing (AST) coverage in regional laboratories, and the percentage of samples with priority resistance genes (e.g., ESBLs) in routine screening. There are many real-world challenges to overcome in order to implement this framework, though. Poor pastoral mobility; dependence on informal, unregulated drug markets; a lack of laboratories in rural parts of the country; and differing regulations in countries make coordinated action difficult.

Thus, a framework for implementation needs to establish clear responsibilities—namely, the regional regulations need to be harmonized along migratory routes, and veterinary extension officers need to adapt educational interventions to mobile communities, while regional laboratories need to be equipped to scale up AST capabilities. International and regional cooperation are vital because the camel trade, migration and the sharing of rangelands may cross national borders [148]. Harmonized regional guidance will enable shared capacity and data exchange in laboratories, enabling surveillance to inform coordinated stewardship, not remain siloed within the different surveillance systems in each country.

## 10. Limitations

There are a number of limitations to this narrative review that should be acknowledged. Firstly, most of the primary pharmacokinetics and pharmacodynamics information for camels is lacking, with much being extrapolated from other camelids and mixed livestock pastoral systems. Moreover, the available literature is geographically limited to the Arabian Gulf, North Africa and East Africa, which might not be a global representation of camel production. Lastly, the genetic association of camel resistance with human clinical infection is still scarce; there is no definitive evidence for the genetic association of camel resistance with human clinical infection, and most of the current One Health transmission risk evidence relies on biologically plausible links and phenotypic similarities.

## 11. Conclusions

Dromedary camels are in a paradoxical situation in the global animal health picture—a growing source of food and livelihood yet, at the same time, peripheral to formal veterinary pharmacology and antimicrobial resistance monitoring. Review of evidence here suggests that the general trend in camel medicine is the off-label and extra-label use of antimicrobials, as there are almost no licensed products that are specific to camel species, pastoral veterinary access is limited, and informal retailing is poorly regulated. These practices result in significant welfare costs, poor management of the infection, inconsistent pain management and risk of under- and overdosing with extrapolated regimens. Clinically relevant resistant and multidrug-resistant bacteria have already been reported from camel milk and feces and ectoparasites from camels throughout the Arabian Gulf, North Africa and East Africa, and there are potential routes to man and the environment. The importance of a One Health perspective is highlighted by the fact that camel health, human health and environmental integrity are inextricably linked. The next steps, which would close the pharmacological evidence gap, improve veterinary access and involve the inclusion of camels in resistance surveillance systems, are achievable; otherwise, the dromedary camel is likely to continue to be both a victim of poor veterinary pharmacology and an under-recognized contributor to the global antimicrobial resistance burden.

## Data Availability

No new data were created or analyzed in this study. Data sharing is not applicable to this article.
